# Supplementary material for: The neural dynamics associated with computational complexity
Source: PLoS Comput Biol. 2024 Sep 23;20(9):e1012447. doi: 10.1371/journal.pcbi.1012447 (PMC11449275; doi:10.1371/journal.pcbi.1012447)
Supplement: S1 Appendix — (PDF) [file pcbi.1012447.s001.pdf]

The neural dynamics associated with computational complexity

S1 Appendix: Supplementary methods and results

Juan P. Franco, Peter Bossaerts, Carsten Murawski*

September 11, 2024

This PDF file includes:

Section 1. fMRI preprocessing.

Section 2. Tables. Human performance in the knapsack decision task.

Section 3. Replication of previous behavioral results. Knapsack optimization and decision tasks.

Section 4. Neural correlates of Instance Complexity (IC).

Section 5. Cognitive function tasks. Correlation between performance on five aspects of cognitive function and performance in the knapsack tasks.

Section 6. Neural correlates of accuracy.

Section 7. Connectivity analysis. Functional and effective connectivity analyses.

1 fMRI preprocessing

1.1 General pipeline

Raw images were organized and converted to the relevant format according to the (BIDS) standards.

Pulse and cardiac noise were regressed out from the functional scans using RETROICOR. These were then slice-time corrected and the volumes were motion-corrected by registering to the first volume of the first functional run. The anatomical (T1) image was down-sampled to the functional EPI resolution ($1.6mm^3$) and the mean BOLD volume of the first run was co-registered to the down-sampled anatomical scan. This transformation was applied to all of the BOLD volumes. Afterwards, each participant's anatomical scan was used for calculation of transformation parameters to normalize the functional images into the Montreal Neurological Institute (MNI) space (Fig A).

Whole-brain analyses were performed by fitting generalized linear models (GLM) using AFNI [1]. Before the regressions were implemented, we spatially smoothed the functional volumes with a 4.8mm FWHM Gaussian kernel. Each voxel's signal was then scaled (per run) to have the same mean (100). Additionally, volumes with motion or signal outliers were censored from each of the regressions. Regressions were performed using the 3dREMLfit algorithm in AFNI. Group level statistical tests were performed using mixed effects multilevel modeling [2].

For the connectivity analyses the volumes were not smoothed, but motion parameters were regressed out before extracting the relevant ROI signals. Whitened (ARMA(1,1)) residuals were used in the subsequent analyses.

1.2 Granger Causality preprocessing: DCM

In addition to the preprocessing steps presented in the previous section, we fitted a dynamic causal model (DCM) before the Granger causality (GC) analysis. This was done in order to remove signals of no interest related to perceptual processes related to screen and stage changes. The model was fit using the SPM12 software (https://www.fil.ion.ucl.ac.uk/spm/software/spm12/) and the residuals of the resulting model were then used to fit the VAR model and test for GC (see section 7.1). In this section we describe the DCM used.

Let z denote a vector of neural activity in 3 regions, indexed i $(= rAI, rAG, dACC)$.

Let v_j denote conditions; they reflect stages in the task conditional on properties of the instance (i.e., satisfiability and TCC). The variable is a dummy variable that takes the value of 1 when the j

Fig A: **fMRI data preprocessing pipeline.** Depiction of the preprocessing steps used prior to the statistical analyses performed on the functional data. The preprocessing steps, up to outlier detection, are shared across all types of analysis. Afterwards, preprocessing steps differ between GLMs and functional connectivity models.

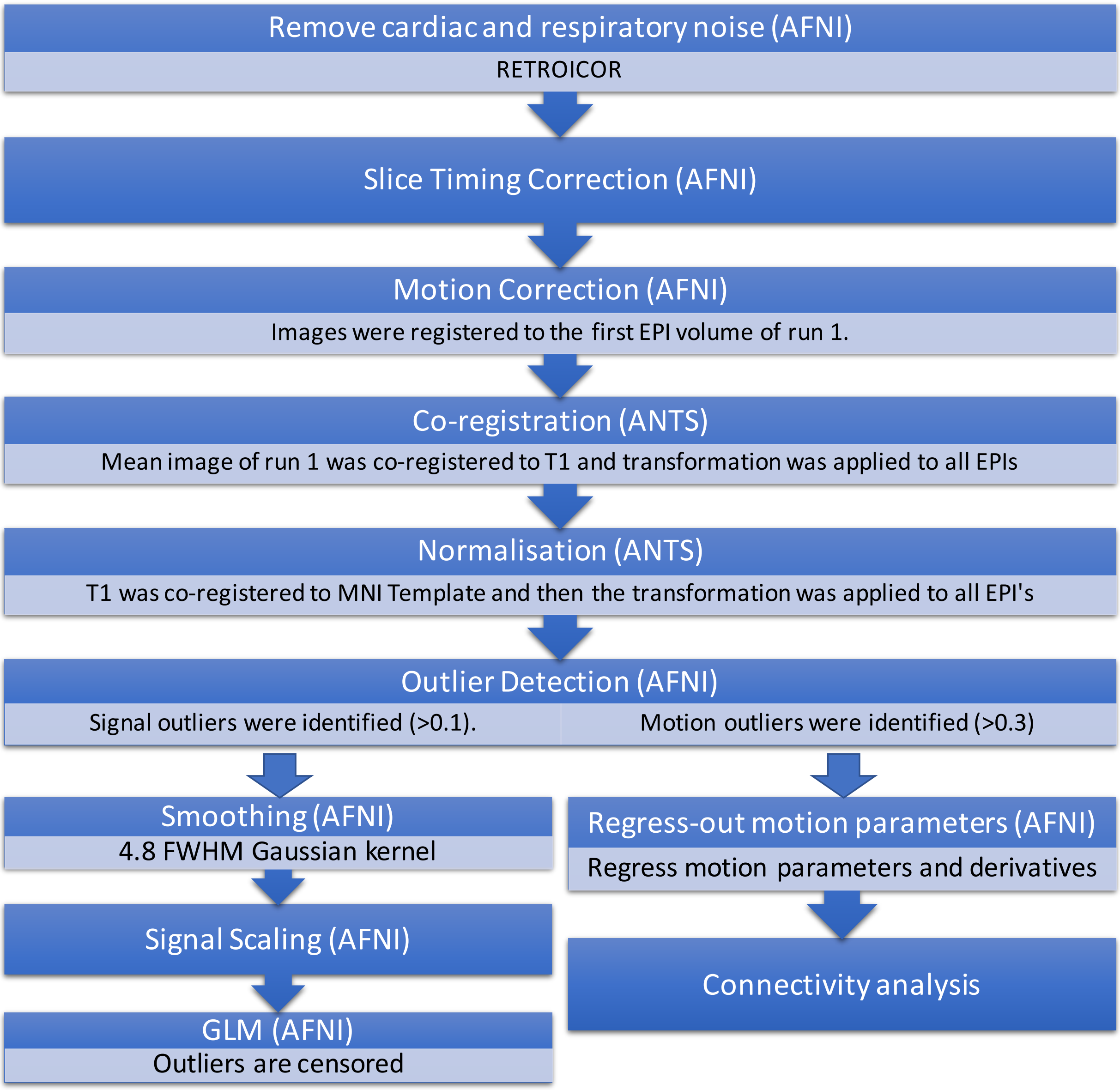

condition is ON the screen:

- $j = 1$: items stage and solving stage (25s),
- $j = 2$: response stage (2s),

Additionally, let o_j denote onsets of conditions; that is, when a stage becomes visible on the screen.

We follow the SPM's notation to describe the model employing three different types of matrices. Matrix A specifies the baseline effective connectivity. Matrix $B^{(j)}$ denotes the modulation of effective

connectivity due to experimental condition j. Finally, $C^{(j)}$ captures the change of the neural response due to the onset of condition j.

The DCM fit is described by the following three equations; one for each ROI:

For rAG:

$$\frac{dz_{rAG}}{dt} = -0.5\exp\left(A_{rAG} + B^{(1)}_{rAG}(u_1)\right) z_{rAG} + \tag{1}$$

$$(B^{(1)}_{dACC\to rAG}(u_1))\, z_{dACC} + (B^{(1)}_{rAI\to rAG}(u_1))\, z_{rAI} + \tag{2}$$

$$+ C^{(1)}_{rAG} o_1;$$

For rAI:

$$\frac{dz_{rAI}}{dt} = -0.5\exp\left(A_{rAI} + B^{(1)}_{rAI}(u_1)\right) z_{rAI} + \tag{3}$$

$$(B^{(1)}_{dACC\to rAI}(u_1))\, z_{dACC} + (B^{(1)}_{rAG\to rAI}(u_1))\, z_{rAG} + \tag{4}$$

$$+ C^{(2)}_{rAI} o_2;$$

As to dACC:

$$\frac{dz_{dACC}}{dt} = -0.5\exp\left(A_{dACC} + B^{(1)}_{dACC}(u_1)\right) z_{dACC} + \tag{5}$$

$$(B^{(1)}_{rAG\to dACC}(u_1))\, z_{rAG} + (B^{(1)}_{rAI\to dACC}(u_1))\, z_{rAI} + \tag{6}$$

$$+ C^{(2)}_{dACC} o_2;$$

It is worth noting the asymmetries between regions in our specification. These are found in the burst of activity in the model (C matrix). Specifically, we expected the AG to be a processing unit with activity starting quickly from the items stage in the task; this is reflected in the $C_{rAG,1}o_1$ term in the rAG equation. In contrast, we expected the AI and dACC to present burst activity related to control and monitoring signals at the moment the solving stage ends (i.e., $C_{rAI,2}o_2$ and $C_{dACC,2}o_2$). Besides this asymmetry, the model allows for a symmetric inter-connectivity between ROIs during the items and solving stage of the task.

2 Tables

Table A: **Human performance in the knapsack decision task.** Logistic regressions with random intercept effects for participants relating the accuracy on an instance and trial number (1), typical-case complexity (TCC) (2), instance complexity (IC) (3), the number of witnesses (4), satisfiability (5), as well as TCC and satisfiability (6). *Parameter estimates correspond to the median of the posterior distribution* ($\beta_{0.5}$) *and the 95% HDI credible interval* ($HDI_{0.95}$)*. ELPD denotes the expected log posterior predictive density.*

	Dependent variable: Human performance					
	(1)	(2)	(3)	(4)	(5)	(6)
Trial Number	0.01<br>[0.00, 0.02]					
TCC		-1.10<br>[-1.44, -0.79]				-1.23<br>[-1.66, -0.79]
IC			6.54<br>[4.67, 8.29]			
No. of Witnesses				0.20<br>[0.12, 0.28]		
Satisfiability					0.02<br>[-0.30, 0.30]	-0.14<br>[-0.61, 0.37]
TCC:Satisfiability						0.26<br>[-0.37, 0.9]
Intercept	1.24<br>[0.83,1.66]	2.05<br>[1.61,2.52]	0.60<br>[0.14,1.03]	0.65<br>[-0.02,1.33]	1.41<br>[1.00,1.81]	2.13<br>[1.63,2.67]
Observations	1120	1120	1120	560	1120	1120
ELPD	-546.77	-523.90	-516.67	-237.33	-548.05	-525.37

3 Replication of previous behavioral results

3.1 Knapsack decision task

An additional aim of this study was to reproduce the key findings presented in [3]. We first looked at the effect of experience on accuracy and found a non-significant improvement as the task progressed ($\beta_{0.5} = 0.009$, $HDI_{0.95} = [-0.001, 0.021]$, main effect of trial number on performance, generalized logistic mixed model (GLMM); Table A Model 1). This marginal improvement in the task performance might seem to contradict previous results, which suggest that neither experience with the task nor mental fatigue affected task performance. However, unlike [3], we performed the task in the scanner, thus this discrepancy could be due to acclimatization to the scanner.

In the main text we show that the results regarding TCC and satisfiability are mirrored by our data and statistical analyses. Specifically, our findings corroborate the significant effect of TCC on performance and replicate a null effect of satisfiability on performance. Additionally, other key findings in their study were related to two *solution-space* metrics of complexity: The number of solution witnesses and instance complexity (IC). The former is defined as the number subsets of items that satisfy both profit and capacity constraints while IC is defined as the distance between the level of the profit constraint (target profit) and the maximum value attainable in the corresponding instance of the optimization variant of the 0-1 knapsack problem. Specifically,

$$IC = \left|\frac{p - p^{\star}}{\sum v_i}\right| = \left|\alpha_p - \alpha_p^{\star}\right|, \tag{7}$$

where $\sum v_i$ is the summation of all item values, p is the target profit of the decision instance, and $p^{\star}$ is the maximum value achievable in the corresponding optimization instance, that is, the maximum value that can be packed into the knapsack given the same set of items I and the same capacity constraint c. α_p and $\alpha_p^{\star}$ denote the normalized values of target profit and optimum value, respectively.

In order to estimate these metrics, unlike TCC, the problem needs to be solved. Concretely, harder versions of the problem need to be solved. For IC to be estimated, the optimization variant of the knapsack problem needs to be solved, while for the number of witnesses all of the possible sets of items that satisfy the constraints need to be found. This makes estimating these metrics more computationally intensive than estimation of TCC. Despite this drawback, these metrics capture the hardness of a single instance of the problem and therefore are more precise when predicting

performance for each instance compared to TCC, which captures the average hardness of an ensemble of random instances.

Franco et al. [3] showed that human performance was affected by both IC and the number of witnesses. Here we reproduced these findings. We found that higher values of IC were related to higher accuracy ($\beta_{0.5} = 6.54$, $HDI_{0.95} = [4.67, 8.29]$, main effect of IC, GLMM; Table A Model 3; Fig B). Similarly, among satisfiable instances, we found that a higher number of witnesses was related to better performance ($\beta_{0.5} = 0.20$, $HDI_{0.95} = [0.12, 0.28]$, main effect of number of witnesses in satisfiable instances, GLMM; Table A Model 4). It is worth noting that the number of witnesses can only explain variability among satisfiable instances since all unsatisfiable instances have 0 witnesses.

Fig B: **Relation between IC and human performance in the knapsack decision task.** Mean accuracy per instance and the marginal effect of IC on human performance (GLMM; Table A Model 3). Higher IC is related to lower computational hardness. Instances are categorized by their TCC (shape) and satisfiability (color).

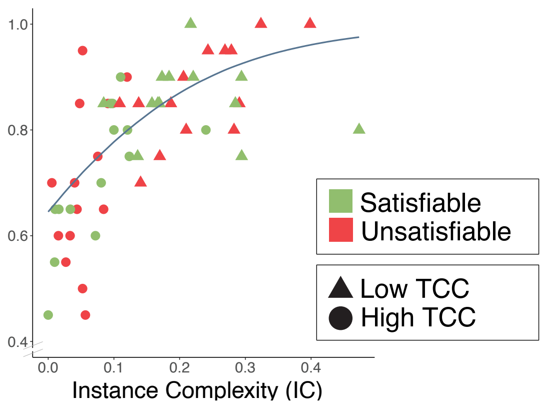

Overall, these results replicate previous findings [3] and validate that the experimentally modulated variable (TCC) successfully varied the computational difficulty of the task.

3.2 Knapsack optimization task

In this task, participants were asked to solve a number of instances of the (0-1) knapsack optimization problem (Fig Ca). In each trial, they were shown a set of items with different weights and values as well as a capacity constraint. Participants had to find the subset of items that maximized total value subject to the capacity constraint. This means that while in the knapsack decision task, participants only needed to determine whether a solution existed, in the knapsack optimization task, they also needed to determine the nature of the solutions (i.e., the items in the optimal knapsack).

For this task we aimed at replicating the results found in [3]. In their study, a metric of

Fig C: **Knapsack optimization task. (a) Experimental design.** Participants were presented with a set of items of different values and weights together with a capacity constraint shown at the center of the screen. The green circle at the center of the screen indicated the time remaining in this stage of the trial. Participants had to find the subset of items with the highest total value subject to the capacity constraint. This stage lasted up to 60 seconds. Participants selected items by clicking on them and had the option of submitting their solution before the time limit was reached. After the time limit was reached or they submitted their solution, a fixation cross was shown for 10 seconds before the next trial started. **(b) TCC_O and human performance.** Human performance corresponds to mean computational performance on each instance. **(c) TCC_O and time-on-task.** Mean time spent before skipping to the response screen. *Each dot represents an instance and is categorized according to its TCC_O. The box-plots represent the median, the interquartile range (IQR) and the whiskers extend to a maximum length of 1.5*IQR*

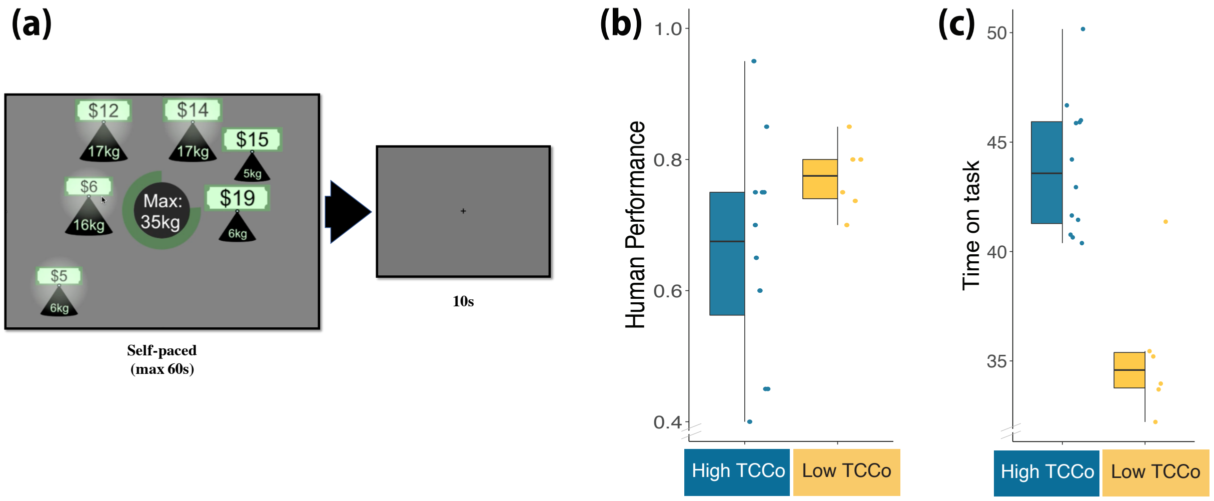

complexity (TCC_O) was introduced as an extension of the TCC metric to optimization problems. Specifically, TCC_O was defined as the TCC of the decision of determining whether the optimal profit (α_p^*) is attainable given the capacity constraint. We expected to replicate the negative effect of TCC_O on performance and its positive effect on time-on-task.

The task consisted of a single solving stage (60 seconds) and an inter-trial interval (fixation cross for 10 seconds). During the solving stage the items and the capacity constraint were presented in the same way as in the knapsack decision task. Unlike in the decision task, however, there was no target profit and participants were able to add and remove items to/from the knapsack by clicking on the items. An item added to the knapsack was indicated by a halo around it (Fig C). Participants could submit their solution before the time limit was reached. If participants did not submit within the time limit, the items selected at the end of the trial were automatically submitted as the solution. Participants were then shown a fixation cross (10 seconds) before the start of the next trial.

Each participant completed 18 trials (2 blocks of 9 trials with a rest period of 60 seconds between blocks). Each trial presented a different instance of the knapsack optimization problem with varying

levels of computational complexity. Specifically, we employed the same instances of the knapsack optimization problem used in [3]. In their study, 12 instances were selected to have high TCC_O and 6 instances were selected to have low TCC_O. All instances had $N = 6$ items and w_i, v_i, c and p were integers. The order of presentation of instances in the task was randomized for each participant. For the analysis, we excluded 1 trial from one participant because solutions were submitted after less than 1 second into the task. Additionally, 2 participants were excluded from the analysis of time-on-task because they never submitted a solution before time ran out.

We investigated two variables related to behavior: performance and time-on-task. Performance was quantified by *computational performance*, which captures participants' ability to find the optimal solution. Specifically, it is defined as a binary variable that is equal to 1 if the participant obtained a value equal to the maximum value obtainable in the instance, and 0 otherwise. It is worth noting that an instance was only characterized as correct if the sum of weights did not exceed the capacity constraint. Mean computational performance was 69.6% (min = 0.18, max = 1, $SD = 0.25$) and the capacity constraint was only violated in 3.9% of instances. Additionally, we investigated time-on-task. In contrast to the decision variant, the optimization task was self-paced and, as such, participants were allowed to submit their answer before the time limit (60s) was reached. We recorded the time participants spent in the solving stage before submitting their candidate solution. Participants spent on average 41.0 seconds on an instance (min = 21.0, max = 55.8, $SD = 8.1$).

We first replicated the effect of trial number in the task. We found that performance did not change throughout the task ($\beta_{0.5} = 0.03$, $HDI_{0.95} = [-0.02, 0.09]$, main effect of trial number on computational performance, GLMM; Table B Model 1), nor did the time-on-task per instance ($\beta_{0.5} = -0.02$, $HDI_{0.95} = [-0.03, 0.22]$, main effect of trial number on time-on-task, CLMM; Table B Model 3). These results suggest, in line with previous results [3], that neither experience with the task nor mental fatigue affected the quality and speed of finding a solution.

Finally, we studied the effect of TCC_O. We expected that performance in instances with *high* TCC_O (instances whose solutions have a corresponding decision problem with high TCC) would be lower than in instances with *low* TCC_O (instances whose solutions have a corresponding decision problem with low TCC). We, indeed find this effect on both computational performance and time-on task. Mean computational performance was lower in instances with high TCC_O, relative to those with low TCC_O ($\beta_{0.5} = -0.75$, $HDI_{0.95} = [-1.35, -0.14]$, main effect of TCC_O on performance, GLMM; Fig Cb; Table B Model 2). Similarly, we found a positive effect of TCC_O on time-on-task

Table B: **Computational performance and time-on-task in the knapsack optimization task.** Models on computational performance represent logistic regressions with random intercept effects for participants. Regression parameters relate performance to trial number (1), and optimization typical-case complexity (TCC_O) (2). Models on time-on-task represent censored linear regressions (with random intercept effects for participants) relating time spent on an instance to trial number (3), and optimization typical-case complexity (TCC_O) (4). *Parameter estimates correspond to the median of the posterior distribution ($\beta_{0.5}$) and the 95% HDI credible interval ($HDI_{0.95}$). ELPD denotes the expected log posterior predictive density.*

	Dependent variable			
	Computational performance		Time-on-task	
	(1)	(2)	(3)	(4)
Trial Number	0.03		-0.02	
	[-0.02,0.09]		[-0.26,0.22]	
TCC_O		-0.75		8.78
		[-1.35,-0.14]		[6.45,10.97]
Intercept	0.84	1.61	41.21	35.12
	[0.05,1.7]	[0.74,2.45]	[36.51,45.47]	[30.98,39.98]
Observations	359	359	323	323
ELPD	-184.19	-181.54	-1228.64	-1199.62

($\beta_{0.5} = 8.78$, $HDI_{0.95} = [6.45, 10.97]$, main effect of TCC_O on time-on-task, CLMM; Fig Cc; Table B Model 4). These results replicate those found in [3].

4 Neural correlates of Instance Complexity (IC)

In section 3.1, we introduced Instance Complexity (IC). This metric is closely related to TCC and captures the computational difficulty of solving the knapsack decision problem. The main advantage of IC over TCC is that it captures the complexity of a single instance of the problem, in contrast to TCC, which captures the average complexity over a collection of instances (a random ensemble of instances with a fixed α). Despite this advantage, our research aim is to explore whether the neural correlates of complexity before the instance is resolved. Therefore, in the main text, we studied the neural correlates of TCC since it can be estimated by simple addition and division without the need for solving the instance; in contrast, IC is as difficult to compute, if not harder, than solving the problem. Accordingly, we expected the neural correlates of IC could predominantly appear later in the task. Despite this limitation, studying the neural correlates of IC could also shed light on the neural processes of complex problem-solving and importantly, help us corroborate the robustness of our findings of the neural correlates of TCC. For this, we explored the parametric modulation of IC in a whole-brain Boxcar analysis (Fig D).

The overall neural correlates found with IC and TCC exhibited considerable similarity (Table C). For period S1, no significant cluster formations were identified when using either IC or TCC. Analysis during the S2 phase indicated two clusters correlated with IC - the Superior Frontal Gyrus (SFG) and the right orbitofrontal cortex. Both clusters have an equivalent cluster in the TCC contrast. Remarkably, in the S3 phase contrast, we identified only a single cluster modulated by IC in the posterior cingulate, but this cluster did not appear when using TCC. The final phase, S4, presented a partial overlap between the neural correlates of IC and TCC. Importantly, these include the right Anterior Insula, right Angular gyrus, and the dorsal Anterior Cingulate Cortex (dACC).

Note that although TCC and IC showed similar activity patterns, there are differences. We observed variations in the specific cluster formations between the two. Interestingly, clusters resulting from TCC were larger compared to those observed when using IC. Also, certain clusters evident with TCC in the S2 and S3 periods were not found when using IC. These variations are potentially due to statistical power. The experimental design was balanced in a way to optimize power to find the categorical effects of TCC and satisfiability, not for the parametric modulation effect of IC.

Overall, the correlates of IC provide further support to the main findings in relation to complexity correlates late in the trial. In particular, in relation to the key ROIs considered for the

Fig D: **Neural correlates of Instance Complexity.** Brain activation effect estimates (β) for the parametric modulation of IC. A positive contrast represents a higher BOLD activity on instances that had lower complexity. Significant cluster-wise FWE-corrected ($p < 0.05$) clusters (with an uncorrected threshold of $p < 0.001$) are presented for each of the contrasts estimated using the Boxcar analysis. Each panel represents a different period in the trial. No significant clusters were found for the contrast during period S1 of the solving stage.

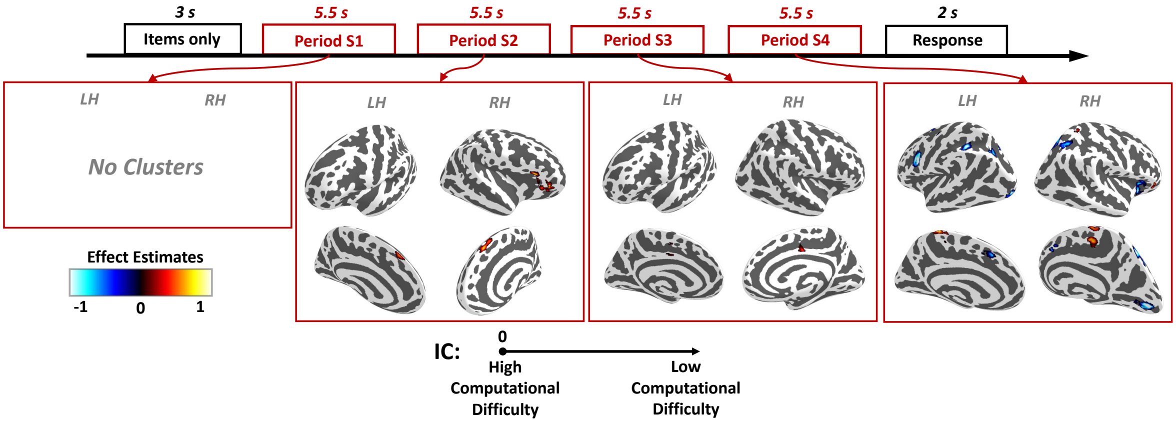

exploration of the temporal dynamics. However, it is worth noting that TCC and IC are highly correlated in this study. Future work could explore whether differences between TCC and IC could dissociate correlates associated with computational work and those associated with subjective neural markers. Designing an experiment with the appropriate instance properties might allow for disentangling these effects.

Table C: **IC clusters**. Significant cluster-wise FWE-corrected ($p < 0.05$) clusters (using an uncorrected threshold of $p < 0.001$) from the parametric modulation of IC. Coordinates are in MNI space.

			Cluster statistics			Peak statistics			
Stage	Region	Side	Volume(mm^3)	β_{mean}	SEM	β_{peak}	x	y	z
S2	Orbitofrontal cortex	RH	2433.0	1.00	0.013	1.86	51	26	-12
	SFG	RH/LH	1142.8	0.84	0.011	1.45	2	44	52
S3	Posterior cingulate	RH/LH	483.3	0.69	0.013	0.94	-3	-9	45
S4	IPS (precuneus)	RH	1380.4	-1.05	0.014	-1.84	13	-76	60
	Precentral gyrus	LH	1220.6	-1.19	0.018	-1.94	-43	4	24
	dACC	LH/RH	1191.9	-0.96	0.009	-1.26	-2	15	47
	Postcentral gyrus	RH/LH	1007.6	0.87	0.017	1.77	3	-31	79
	Occipital superior cortex	LH	843.8	-0.91	0.016	-1.48	-37	-66	60
	Fusiform gyrus	LH	741.4	-0.81	0.008	-1.08	-24	-81	-9
	Superior parietal lobule	RH	659.5	0.81	0.012	1.20	40	-44	68
	Intracalcarine	RH	651.3	-1.08	0.019	-1.49	13	-82	8
	Precentral gyrus	RH	630.8	0.65	0.008	0.87	6	-31	50
	AI	RH	598.0	-0.90	0.015	-1.41	32	23	-4
	AG	RH	598.0	-1.05	0.015	-1.46	30	-66	47
	Orbitofrontal cortex	RH	569.3	0.99	0.013	1.54	53	31	-14
	Supramarginal gyrus	LH	536.6	-1.04	0.013	-1.34	-40	-42	37
	MFG/SFG	LH	528.4	-0.93	0.012	-1.40	-29	-1	61
	Occipital Pole	LH	528.4	-1.08	0.012	-1.20	-38	-94	-12

5 Cognitive function tasks

In a previous study, we tested participants' performance on five aspects of cognitive function that we considered relevant for the knapsack tasks [3]. Explicitly, we assessed working memory, episodic memory, strategy use, processing and psychomotor speed, as well as mental arithmetic. We were interested in finding links between these cognitive capacities and the ability to solve the knapsack task. A complex task that would arguably require the deployment of these other, more basic, cognitive abilities. Our original study lacked the power to identify reliable correlations between performance in these cognitive tasks and performance in the knapsack tasks.

In this study, we tested participants on the same five aspects of cognitive function with the aim of increasing the power of these exploratory tests. For this purpose, we aggregated the data collected in this study with that collected in [3] and estimated the same correlations presented there.

Following the approach in [3] we administered a set of tasks from the Cambridge Neuropsychological Test Automated Battery (CANTAB; [4]). Specifically, we asked participants to perform the Paired Associates Learning (PAL), Spatial Working Memory (SWM) and Spatial Span (SSP). Additionally, participants solved a set of mental arithmetic problems [5]. Below we describe each of the tests performed:

Paired Associates Learning (PAL) Boxes are displayed on the screen and open one by one in a randomized order to reveal patterns hidden inside. The patterns are then displayed in the middle of the screen, one at a time, and the subject must touch the box where the pattern was originally located.

Spatial Working Memory (SWM) The test begins with colored boxes being shown on the screen. The aim of this test is that, by touching the boxes and using a process of elimination, the subject should find one 'token' in each of the boxes and use them to fill up an empty column on the right hand side of the screen. The computer will never hide a token in the same colored box, so once a token is found in a box the participant should not return to that box to look for another token.

Spatial Span Task (SSP) White squares briefly change color in a variable sequence. The participant must remember the sequence and then touch the squares in that same order. The sequence length increases through the test. There are up to 3 attempts at each sequence length and

the test terminates if all three are failed.

Mental Arithmetic Task Participants were asked to answer a set of 33 mental arithmetic problems. They were given 13 seconds to solve each problem. The task involved addition and division of numbers, as well as questions in which they were asked to round to the nearest integer the result of an addition or division operation.

From performance in these tasks we estimated five metrics of cognitive capacities and estimated their correlation with participant's performance on the knapsack decision and optimization tasks. Results are presented in Table D. We found, after correcting for multiple comparisons using Holm-Bonferroni correction, a significant positive effect between performance in the knapsack optimization task and performance in the mental arithmetic task ($\rho = 0.617$ at FWE-corrected $\alpha = 0.05$). Additionally, we found (at FWE-corrected $\alpha = 0.10$) a negative correlation between the *strategy use* metric and performance in the knapsack decision task ($\rho = -0.421$). The SWMS metric encodes the number of times a subject begins a new search pattern from the same box they started with previously in the SWM task. Therefore, a lower score is interpreted as higher strategy use (1 = they always begin the search from the same box). These results suggest that participants that use a planned strategy in SWM perform better in the knapsack decision task.

Table D: **Pearson correlations between performance in the knapsack tasks and cognitive abilities.** Performance in the knapsack decision task is characterized by accuracy and in the knapsack optimization task is characterized by computational performance. The cognitive abilities measured used were mental arithmetic, episodic memory (PALFAMS28), working memory (SSPFSL), strategy use (SWMS) and spatial working memory (weighted SWMTE, with errors on easier tasks being weighted more). Standard errors are shown in parentheses. P-values are shown without multiple comparisons correction. [1]In the mental arithmetic task $df = 37$. *Note: FWE significance* * *<0.1;* ** *<0.05;* *** *<0.01 is assessed employing Holm-Bonferroni correction.*

Task	Knapsack decision	Knapsack optimization
Mental arithmetic	0.311<br>(0.156)<br>p=0.054	0.617***<br>(0.129)<br>p=0.000
Episodic memory	0.098<br>(0.161)<br>p=0.549	-0.017<br>(0.162)<br>p=0.918
Working memory	0.033<br>(0.162)<br>p=0.839	0.218<br>(0.158)<br>p=0.177
Strategy use	-0.421*<br>(0.147)<br>p=0.007	-0.336<br>(0.153)<br>p=0.034
Spatial working memory	-0.360<br>(0.151)<br>p=0.023	-0.348<br>(0.152)<br>p=0.028
Degrees of freedom[1]	38	38

6 Neural correlates of accuracy

It has been hypothesized that FPN as well as CON regions encode task signals related to error detection and error expectation [6, 7, 8]. Although participants did not receive any feedback during the task, we expected to see error-related signals during later stages of the trial. Although these signals would not represent the integration of novel exogenous information (since there was no feedback) we conjectured that participants would represent a subjective belief on the expected accuracy (or reward) of their answer [e.g, 9].

We found only one significant cluster during the solving stage (in period one) (Fig Ea; Table E). The other significant clusters were identified during the response stage (Fig Eb; Table E). In line with our hypothesis, during the response stage we found that activity in both the FPN and CON was positively correlated with erring. Specifically, a higher activity was found for incorrect trials in the AI (bilaterally), dACC, left MFG and the right inferior frontal gyrus. Additionally to these regions, which are commonly associated with the multiple-demand system, we also found significant activation in the SFG (bilaterally), ACC and paracingulate gyrus.

Fig E: **Neural correlates of accuracy.** Brain activation effect estimates (β) for the correct vs. incorrect contrast ($\beta_{correct} - \beta_{incorrect}$). A positive contrast represents a higher BOLD activity on instances that were answered correctly. Significant cluster-wise FWE-corrected ($p < 0.05$) clusters (with an uncorrected threshold of $p < 0.001$) are presented for each of the contrasts estimated using the Boxcar analysis. Each panel represents a different period in the trial. **(a)** Period S1, **(b)** response stage. No significant clusters were found for the contrasts during periods S2-S4 of the solving stage.

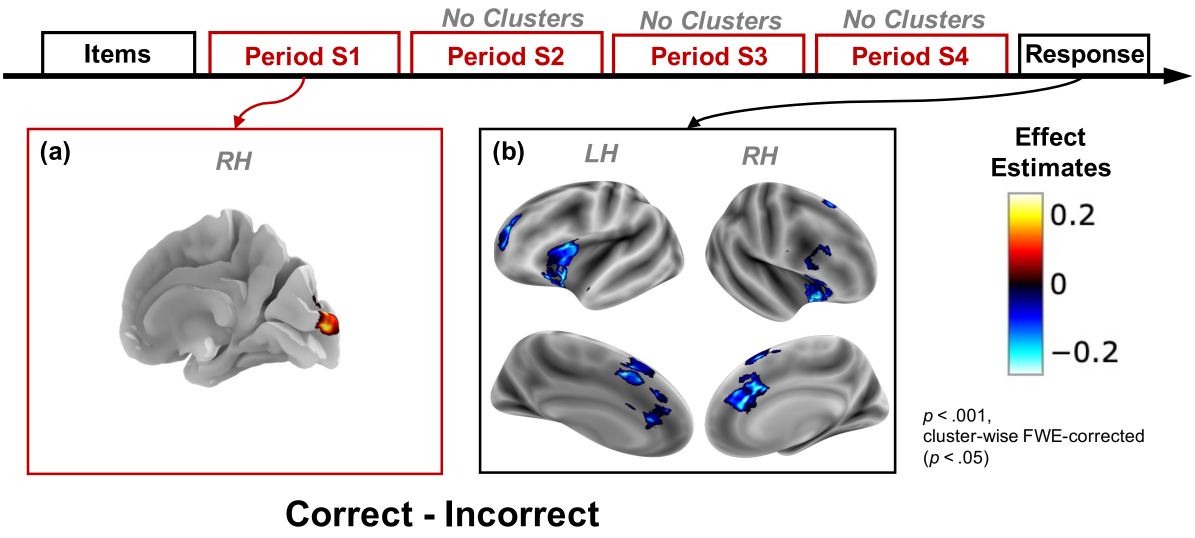

The results of this analysis confirm our hypothesis by outlining a set of regions in both FPN and CON that encode errors during the response stage. In contrast, this analysis did not result in any other clusters during the solving stage that correlated negatively with accuracy. The lack of negative

Table E: **Accuracy clusters**. Significant cluster-wise FWE-corrected ($p < 0.05$) clusters (using an uncorrected threshold of $p < 0.001$) from the *Correct-Incorrect* contrast. Coordinates are in MNI space.

			Cluster statistics			Peak statistics			
Stage	Region	Side	Volume(mm^3)	β_{mean}	SEM	β_{peak}	x	y	z
S1	Occipital cortex	RH	569.3	0.21	0.003	0.31	10	-100	8
Response	AI	RH	2146.3	-0.22	0.002	-0.33	30	23	-8
	AI	LH	2048.0	-0.26	0.002	-0.40	-48	18	-12
	dACC	LH	1953.8	-0.24	0.002	-0.38	-2	22	40
	SFG	RH	1007.6	-0.18	0.003	-0.28	2	23	60
	MFG	LH	974.8	-0.19	0.002	-0.27	-27	50	16
	SFG	LH	684.0	-0.18	0.003	-0.26	-2	10	60
	Inferior frontal gyrus	RH	602.1	-0.22	0.003	-0.29	50	17	31
	ACC	LH	520.2	-0.18	0.002	-0.25	-3	31	26
	Paracingulate gyrus	LH	491.5	-0.24	0.004	-0.31	-5	9	50

correlation during the late periods of the solving stage could be due to variability in the signal during the solving stage. Indeed, during this stage participants might be updating their accuracy expectation as well as their candidate response. Since our accuracy contrast is based on the answer provided during the response stage, it stands to reason that our analysis does not capture accuracy markers during the solving stage because we do not have a measure of accuracy during this period. It is worth noting that we found one significant cluster during the solving stage (in period one) that correlated positively with accuracy in the occipital cortex. This could reflect attentional differences, early in the trial, which affect performance on the trial.

7 Connectivity analysis

Connectivity analysis was performed over the three considered ROIs (dACC, rAG and rAI). To remove non-neural sources from the neural signal, the motion parameters were regressed out before extracting the relevant ROI signals. We then performed connectivity analysis using two separate approaches.

Psychophysiological interactions (PPI)

To study the effect of TCC and satisfiability on *functional connectivity*, we conducted a PPI analysis to gauge the functional synchronization between each of the ROIs (as seed regions) and other regions in the brain. For these regressions, we modeled the task (items and solving stages together) with two boxcar functions of equal length (12.5s) (Fig G). This allowed us to study PPI task interactions separately for an early period (PPI-1: first 12.5 seconds of the task) and a late period (PPI-2: last 12.5 seconds).

We performed generalized PPI analyses using AFNI. We ran two separate regressions for each ROI; one for satisfiability and one for TCC. Each PPI regression was estimated according to the following:

$$y = \beta_0 + \beta_1 S_{ROI} + \beta_3 L_0 \times box_{PPI1} + \beta_4 L_1 \times box_{PPI1} + \beta_5 L_0 \times box_{PPI2} + \beta_6 L_1 \times box_{PPI2} + \beta_7 L_0 \times box_{PPI1} \times S_{ROI} + \beta_8 L_1 \times box_{PPI1} \times S_{ROI} + \beta_9 L_0 \times box_{PPI2} \times S_{ROI} + \beta_{10} L_1 \times box_{PPI2} \times S_{ROI}$$

where L_0 corresponds to low TCC (or satisfiable) condition and L_1 corresponds to high TCC (or unsatisfiable) condition. S_{ROI} is the neural signal of the seed region and box_i corresponds to a boxcar function that separates the items and solving stages, together, into two boxcar functions (PPI-1 and PPI-2) of the same duration (12.5s each; Fig G). Note that these boxcar functions are different in duration to the ones used for the boxcar GLM analysis. The contrasts of interest (β_8, β_7, β_{10} and β_9) captured the PPI effects; that is, the task-dependent connectivity to the ROIs for each of the two periods considered. Additionally, we tested whether there were regions that showed a differential connectivity to an ROI between conditions (i.e., high vs. low TCC, unsatisfiable vs. satisfiable). Explicitly, we performed group level analysis using mixed effects multilevel modeling [2] on the contrasts corresponding to $L_1 - L_0$ ($\beta_8 - \beta_7$ and $\beta_{10} - \beta_9$). Results are reported with a clusterwise threshold of $p < 0.05$ corrected for multiple comparisons across the whole brain, using an

uncorrected voxelwise threshold of $p < 0.001$.

It is worth noting that the interaction between box-car functions and the seed region ($box \times S$) was estimated via deconvolution. That is, the BOLD time series of each seed region was deconvolved with a canonical HRF (AFNI: *BLOCK(0.1,1)*) and then multiplied with the psychological boxcar function. This was convolved back with the same HRF to form a predicted PPI time series at the hemodynamic response level (BOLD), at which the regression takes place.

We found a similar and generalized pattern of connectivity for all three ROIs and both periods when contrasting the PPI effect compared to baseline (Fig F). This suggests that the task has a similar effect on the BOLD synchronization between the three ROIs and several regions.

When comparing the connectivity between instances with high and low TCC, we found one significant cluster with differential connectivity. This cluster, located along the rAG and the supramarginal gyrus, showed a change in connectivity to the rAI (seed region) between high and low TCC instances during the second PPI period (Fig Ga; Table F). We also explored the differences in the PPI connectivity between unsatisfiable and satisfiable instances. We observed a significant PPI effect of satisfiability between the right IPS/AG (seed) and the left MFG, as well as with the left AG, during the second PPI period (Fig Gb; Table F). Overall, these results suggest that instance properties have an effect on the synchronicity between the ROIs and a limited collection of clusters. However, this effect is only significant during the later part of the solving stage. PPI analysis, however, only explores temporal connectivity. A closer inspection of the time courses in the ROI dynamics presented in the main text suggests that there may be inter-temporal relationships in activation. We turn to a study of those next.

Table F: **PPI clusters**. The effect of instances' properties on connectivity. Significant cluster-wise FWE-corrected ($p < 0.05$) clusters (using an uncorrected threshold of $p < 0.001$). Coordinates in MNI space.

			Cluster statistics			Peak statistics			
Contrast	Region	Side	Volume(mm^3)	β_{mean}	SEM	β_{peak}	x	y	z
TCC	AG/Supramarginal Gyrus	RH	573.4	0.26	0.004	0.36	61	-50	36
Satisfiability	MFG	LH	499.7	0.300	0.007	0.475	-43	18	59
	AG	LH	483.3	0.266	0.006	0.432	-54	-65	47

Fig F: **PPI supplementary results.** The effect of the task on the connectivity to each of the three seed regions used for the analysis (dACC, rAG and rAI). Each column shows the PPI effect for a different seed region. Each row displays the period of the task considered. Activation patterns represent the significant effect estimates for the PPI on instances with low TCC. The effect for instances with high TCC is not displayed, but the difference with respect to the activation signatures shown here are small. Indeed, the only significant differences between conditions are presented in figure Ga. *Significant cluster-wise FWE-corrected ($p < 0.05$) clusters (with an uncorrected threshold of $p < 0.001$) are presented.*

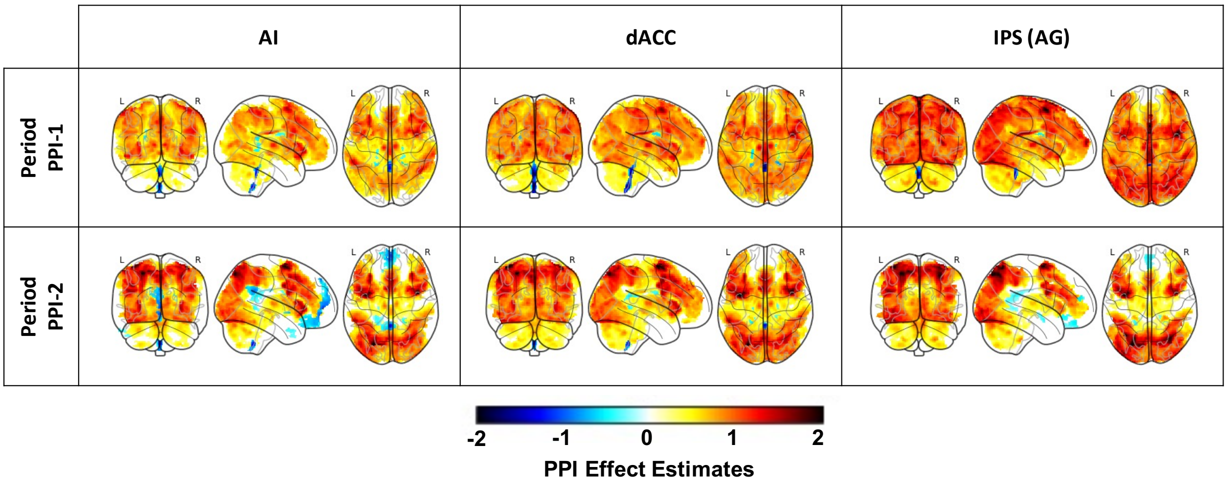

Fig G: **PPI results.** The effect of instance properties on connectivity: TCC and Satisfiability. The left panel represents the seed region used for the analysis (dACC, rAG or rAI). The right panel shows the clusters that display a significant PPI connectivity effect for a particular seed region and period. *Significant cluster-wise FWE-corrected ($p < 0.05$) clusters (with an uncorrected threshold of $p < 0.001$) are presented. "No clusters": No significant clusters were found for this analysis.*

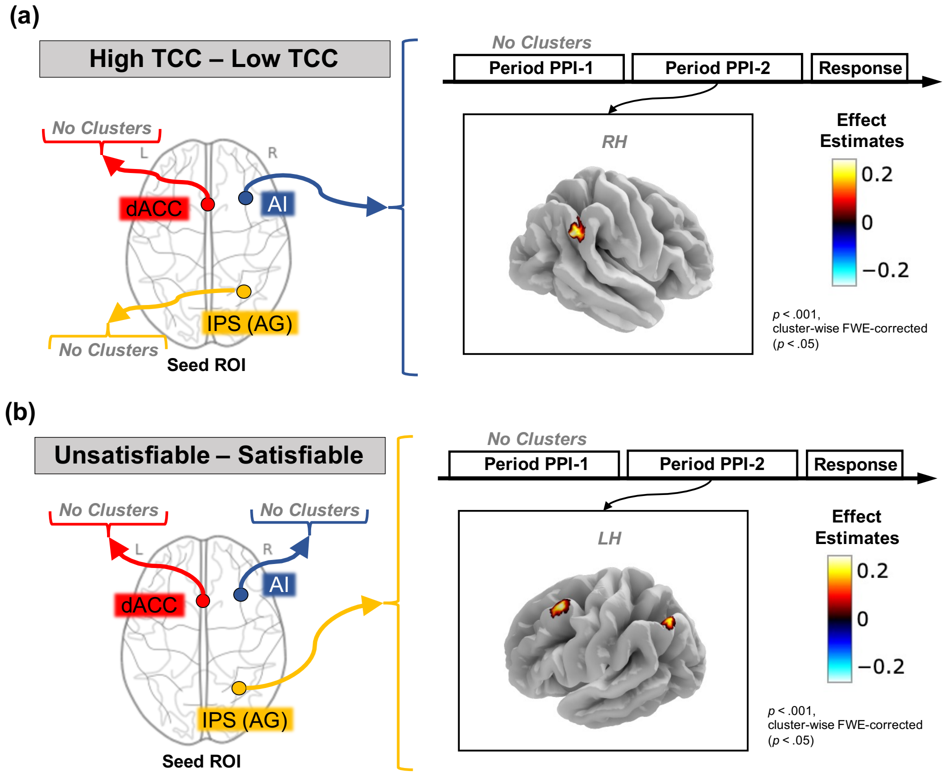

7.1 Granger causality analysis

PPI analysis provides a description of the functional connectivity (synchronization) between regions based on correlations between simultaneous activity across regions. As such, this analysis is insensitive to temporal directionality in the time series. In contrast, Granger Causality (GC) is defined based on Vector Auto Regression (VAR) models, whereby a vector of ROI signals is driven by a finite number of lags of itself. This allows for the gradual excitatory (or inhibitory) impact of one region onto another, which might suggest temporal directionality. This directional effect can be summarized by GC, which emerges when the presence of lags of one variable significantly improves the fit (maximum likelihood value) of another variable.

To perform GC, we first fitted a DCM to the BOLD time series of the three ROIs of interest. This was done to ensure that the DCM captured all the task-relevant events and controls not strictly related to the internal solving process itself (e.g., onset of decision screen). We report the exact specification of the DCM in section 1.2. We then extracted the residual series of the DCM model for each region. We refrained from deconvolving the BOLD residuals (in accordance with [10]) because deconvolution is a smoothing operation that introduces spurious lead-lag relationships.

GC emerges when lagged outcomes of a variable *correlate* significantly with values of another variable. As such, GC is closely linked to *cross-autocorrelations*. Typically, GC is analyzed in the context of a Vector Auto Regression (VAR), i.e., a model whereby a vector of outcomes is driven by a finite number of lags of itself. GC emerges when the presence of lags of one variable significantly improves the fit (maximum likelihood value) of another variable. If this is the case, the former "Granger causes" (GCs) the latter. We ran a VAR on the error series augmented with the error series during the solving stage only, and determined incremental GC of one series on another during problem-solving.

In order to reach a GC statistic at the group level we carried out the following procedure. We first ran a VAR for each subject. Each subject's VAR maximum lag was determined by comparing AIC (Akaike Information Criterium) for lags up to 10. From each regression we extracted 5 GC statistics for each ROI: 2 GCs from lagged time series of each of the other two ROIs and 3 GCs (one for each ROI) from the lagged time series of the solving stage. This process generates 15 GC statistics per subject. To correct for multiple comparisons among these we performed standard Bonferroni correction.

To determine statistical significance at the group level, a standard binomial test was then employed to determine the significance of the frequency of rejections (of no GC) across the 20 participants. A p level of was 0.05 used. FWE correction was applied using Holm-Bonferroni correction over the 15 tests.[1] The Matlab method `gctest` was used to implement the Granger Causality estimations.

Critical for this study, we expected the underlying neural processes of problem-solving to be internally driven. Specifically, we expected the connectivity patterns to be linked to neural processes whose timing could vary stochastically across trials and participants (e.g., the burst of neural activity does not have to coincide with an experimental intervention such as the initial display of items). In order to explore these connectivity patterns we performed a GC analysis on the three ROIs. For this, we ran a VAR model on the ROI time series augmented with the series during the solving stage only, and determined incremental GC of one series on another during problem-solving. This allowed us to estimate effective connectivity GC tests at baseline as well as the GC changes from baseline during the solving stage.

Our analysis shows differential activation between baseline and solving stage. At baseline, during the experiment, we find a bidirectional connectivity between all the ROIs (Fig Ha), which then changes during the solving stage. Specifically, there is a significant change in GC from dACC to rAG. In other words, the dACC (lagged time series) effect on rAG is different between baseline and solving stage. Moreover, we find that during the solving stage there was a significant change in self-activation effect in the dACC and rAG; that is, the lagged time series of each of these two ROIs Granger-cause themselves differentially during the solving stage compared to baseline (Fig Hb).

Employing the same methodology, we explored whether the change in effective connectivity between solving stage and baseline is modulated by complexity and proof hardness. Specifically, we explored the effects of TCC and satisfiability on GC. We did not find any significant changes in the effective connectivity between high and low TCC instances (all uncorrected p-val>0.264) nor between unsatisfiable and satisfiable instances (all uncorrected p-val>0.075).

Overall, we found that there was a change in effective connectivity from dACC to IPS during the solving stage of the task. These results extend those previously found in perceptual tasks [11], in

[1] Significance is determined as follows: order p values from small ($k = 1$) to large ($k = 15$); the kth test value is deemed to be significant at the level α if $p(k) \leq \alpha/(m + 1 - k)$ where m is the number of hypotheses to be tested; here: $m = 15$. If $\alpha = 0.05$ then the smallest p should be ≈ 0.0033 for the corresponding test (i.e., the test with smallest p value) to reject.

Fig H: **Granger causality results.** Effective connectivity estimated via Granger causality between each of three ROIs: dACC, rAI and rAG. **(a)** Represents the baseline connectivity between the regions. **(b)** Represents the changes in effective connectivity during the solving stage compared to baseline. Only three effects survive multiple comparisons correction: An increased connectivity from dACC to rAG and a higher self-modulatory effect on both dACC and AG. *P-values correspond to the GC test uncorrected for multiple comparisons. Asterisks represent significant GC effects FWE-corrected at significance threshold of 0.05.*

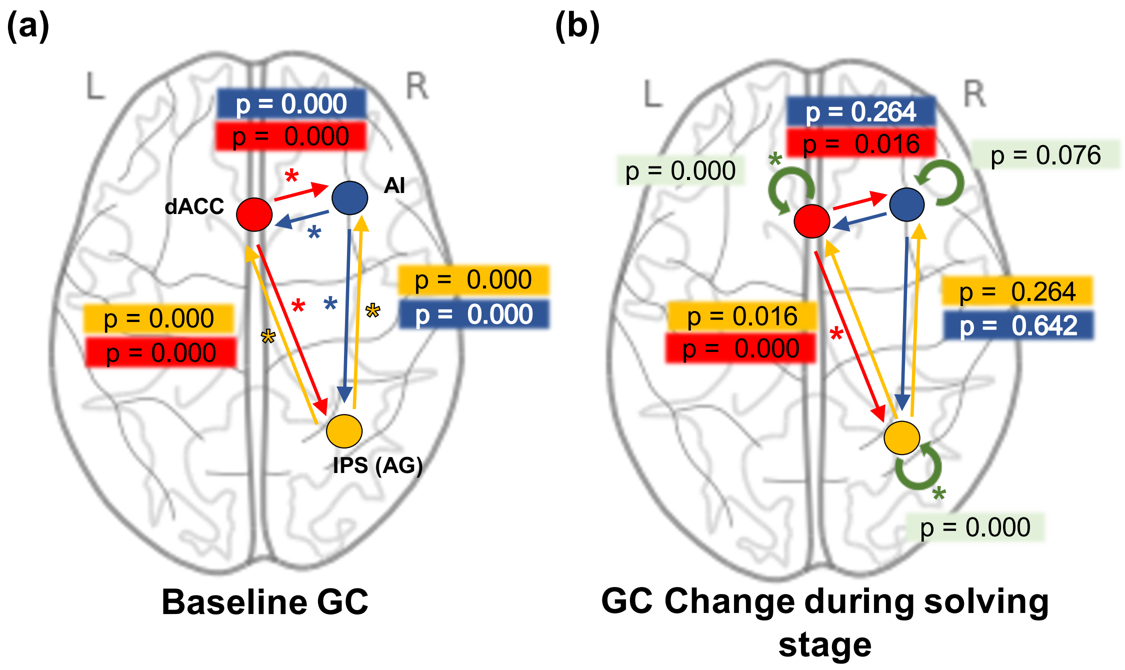

which regions relevant for the task at hand showed a higher functional connectivity to the dACC during the task. This result further supports previous research that assigns to the dACC a central role in the allocation of control [12, 8, 13, 14, 15, 16, 17, 11, 18]. Interestingly, we did not find a significant change in the effective functional connectivity between rAI and rIPS during the solving stage. These finding match previous research that support a dissociation between dACC and AI [19, 20, 21, 22]. However, these results seem to be contrary to those found in [17], where they found increased functional connectivity between AI and task-relevant regions in perceptual and episodic memory tasks. Several possible explanations could be put forward to account for this discrepancy. For instance, the nature of the functional connectivity between rAI and task relevant regions might be task-specific. Specifically, it has been suggested that AI is predominantly involved in processing of internal visceral and motivational information involved in autonomic behavior [20]. This type of processing might be more relevant in perceptual and episodic memory tasks compared to the knapsack task, in which mathematical calculations might be more pertinent. Alternatively, other possible explanations for the lack of significant effective connectivity between rAI and rIPS include

the lack of statistical power in this analysis, as well as discrepancies in the ROI definition.

Another significant aspect of effective connectivity considered was its link to the intrinsic properties of the problem at hand. Our results suggest that the effective connectivity pattern was impervious to the level of computational demand and satisfiability. Of particular relevance, we found that the effective connectivity between dACC and IPS was not modulated by TCC. This suggests that the effect of TCC on control, if any, occurs by generating differential levels of activity within the regions of interest and not via modulation of the functional connectivity between these regions. It is worth noting that this failure to reject the null hypothesis could be due to a lack of power or the exclusion of relevant ROIs from the analysis. We leave it to future research to explore how whole brain connectivity patterns are affected by computational demand.

References

1. Cox RW. AFNI: Software for analysis and visualization of functional magnetic resonance neuroimages. Computers and Biomedical Research. 1996;29(3):162–173. doi:10.1006/cbmr.1996.0014.

2. Chen G, Saad ZS, Nath AR, Beauchamp MS, Cox RW. FMRI group analysis combining effect estimates and their variances. NeuroImage. 2012;60(1):747–765. doi:10.1016/j.neuroimage.2011.12.060.

3. Franco JP, Yadav N, Bossaerts P, Murawski C. Generic properties of a computational task predict human effort and performance. Journal of Mathematical Psychology. 2021;104:102592. doi:10.1016/j.jmp.2021.102592.

4. Cognition C. Cambridge Neuropsychological Testing Automated Battery (CANTAB) [Cognitive assessment software].; 2017. Available from: www.cantab.com.

5. Cappelletti M, Butterworth B, Kopelman M. Spared numerical abilities in a case of semantic dementia. Neuropsychologia. 2001;39:1224–1239.

6. Neta M, Nelson SM, Petersen SE. Dorsal Anterior Cingulate, Medial Superior Frontal Cortex, and Anterior Insula Show Performance Reporting-Related Late Task Control Signals. Cerebral cortex. 2017;27(3):2154–2165. doi:10.1093/cercor/bhw053.

7. Neta M, Schlaggar BL, Petersen SE. Separable responses to error, ambiguity, and reaction time in cingulo-opercular task control regions. NeuroImage. 2014;99:59–68. doi:10.1016/j.neuroimage.2014.05.053.

8. Dosenbach NUF, Visscher KM, Palmer ED, Miezin FM, Wenger KK, Kang HC, et al. A Core System for the Implementation of Task Sets. Neuron. 2006;50(5):799–812. doi:10.1016/j.neuron.2006.04.031.

9. Duverne S, Koechlin E. Rewards and Cognitive Control in the Human Prefrontal Cortex. Cerebral Cortex. 2017;27(10):5024–5039. doi:10.1093/cercor/bhx210.

10. Seth AK, Chorley P, Barnett LC. Granger causality analysis of fMRI BOLD signals is invariant to hemodynamic convolution but not downsampling. NeuroImage. 2013;65:540–555. doi:10.1016/j.neuroimage.2012.09.049.

11. Aben B, Calderon CB, van den Bussche E, Verguts T. Cognitive effort modulates connectivity between dorsal anterior cingulate cortex and task-relevant cortical areas. Journal of Neuroscience. 2020;40(19):3838–3848. doi:10.1523/JNEUROSCI.2948-19.2020.

12. Shenhav A, Botvinick MM, Cohen JD. The expected value of control: An integrative theory of anterior cingulate cortex function. Neuron. 2013;79(2):217–240. doi:10.1016/j.neuron.2013.07.007.

13. Silvetti M, Vassena E, Abrahamse E, Verguts T. Dorsal anterior cingulate-brainstem ensemble as a reinforcement meta-learner. PLoS Computational Biology. 2018;14(8):e1006370. doi:10.1371/journal.pcbi.1006370.

14. Vassena E, Holroyd CB, Alexander WH. Computational models of anterior cingulate cortex: At the crossroads between prediction and effort. Frontiers in Neuroscience. 2017;11(JUN):1–9. doi:10.3389/fnins.2017.00316.

15. Holroyd CB, Yeung N. Motivation of extended behaviors by anterior cingulate cortex. Trends in Cognitive Sciences. 2012;16(2):122–128. doi:10.1016/j.tics.2011.12.008.

16. Alexander WH, Brown JW. Medial prefrontal cortex as an action-outcome predictor. Nature Neuroscience. 2011;14(10):1338–1344. doi:10.1038/nn.2921.

17. Sestieri C, Corbetta M, Spadone S, Romani GL, Shulman GL. Domain-general signals in the cingulo-opercular network for visuospatial attention and episodic memory. Journal of Cognitive Neuroscience. 2014;26(3):551–568. doi:10.1162/jocn_a_00504.

18. Crottaz-Herbette S, Menon V. Where and when the anterior cingulate cortex modulates attentional response: Combined fMRI and ERP evidence. Journal of Cognitive Neuroscience. 2006;18(5):766–780. doi:10.1162/jocn.2006.18.5.766.

19. Han SW, Eaton HP, Marois R. Functional Fractionation of the Cingulo-opercular Network: Alerting Insula and Updating Cingulate. Cerebral Cortex. 2019;29(6):2624–2638. doi:10.1093/cercor/bhy130.

20. Nelson SM, Dosenbach NUF, Cohen AL, Wheeler ME, Schlaggar BL, Petersen SE. Role of the anterior insula in task-level control and focal attention. Brain Structure and Function. 2010;214(5-6):669–680. doi:10.1007/s00429-010-0260-2.

21. Menon V, Uddin LQ. Saliency, switching, attention and control: a network model of insula function. Brain structure & function. 2010;214(5-6):655–667. doi:10.1007/s00429-010-0262-0.

22. Wu T, Wang X, Wu Q, Spagna A, Yang J, Yuan C, et al. Anterior insular cortex is a bottleneck of cognitive control. NeuroImage. 2019;195:490–504. doi:10.1016/j.neuroimage.2019.02.042.
