# Supplementary material for: The neural dynamics associated with computational complexity
Source: PLoS Comput Biol. 2024 Sep 23;20(9):e1012447. doi: 10.1371/journal.pcbi.1012447 (PMC11449275; doi:10.1371/journal.pcbi.1012447)
Supplement: Attachment — Submitted filename: Franco-response_letter.pdf [file pcbi.1012447.s002.pdf]

Dear Editor,

Thank you for considering our revised manuscript, “The neural dynamics associated with computational complexity" (PCOMPBIOL-D-23-01530) for publication in PLOS Computational Biology. We are pleased that the reviewers found our study relevant and interesting. We would like to thank the reviewers for their thoughtful and constructive comments, which have helped us improve significantly. We have addressed all of the issues raised by the reviewers in the attached revisions. All reviewer comments are below in black with our comments in blue.

Reviewer #1:

In this study, the author study the neural correlates of computational hardness. They use the knapsack problem in which a solution is searched to satisfy a set of constraint. Computational difficulty was manipulated by comparing problems that are satisfiable and unsatisfiable (the latter being harder to demonstrated because it is necessary to demonstrate that no solution exist, whereas finding only one solution demonstrates satisfiability). They also manipulated difficulty by manipulating the likelihood of a random instance of the problem satisfying the constraint, which they term “typical case complexity”. They find that (human) subjects’ performance decreases with typical case complexity, but does not change depending on satisfiability. At the neural level, they find that typical case complexity and unsatisfiability correlate with increased activity in a number of regions, including the dorsal anterior cingulate cortex, anterior insula and intra parietal sulcus. They show that these effects typically build up over time within a given problem solving period. They report that these two factors also modulate connectivity between these regions.

This study makes a useful contribution in uncovering some aspects of reasoning in complex task. It benefits from a computational characterization of the task and interesting behavioral results (but see also my concerns below). It is clearly written, thoroughfully analyzed (see the impressive number of appendices), it uses a variety of methods (neural correlates, analysis of temporal dynamics, change in temporal connectivity and directed connectivity), and the methods are generally solid. I list a number of concerns that could improve the paper if addressed in a revision.

Major concerns:
1) Behavioral relevance of satisfiability
The main section reports that there is no behavioral effect of satisfiability, but some neural effects of satisfiability. It would be more convincing if the authors could show some behavioral effect of satisfiability, which would indicate that this concept accounts for the way human subjects solve the task. If I understood correctly, it should take longer to answer the problem when it is not satisfiable, because when it is, it suffices to find one solution to conclude that the problem is satisfiable. In some self-pace version of the task, a difference could be found in reaction times. I apologize if this effect is reported in appendix (I looked for it…) or in a previous publication; in either case it would be useful to draw the attention of the reader to it.

We thank the reviewer for raising this point and allowing us to clarify. The behavioural data we collected was the type of response (satisfiable/unsatisfiable). Based on response type, we determined whether a response was correct or not. As the reviewer points out, we find no differences in correctness between satisfiable and unsatisfiable instances, that is, correctness of responses was not affected by satisfiability. At the same time, we do find differences in neural activation patterns between satisfiable and unsatisfiable instances. This is not surprising given that these instances were of a different nature and required different types of responses (satisfiable/unsatisfiable). In other words, given that participants were able, in many cases, to correctly discriminate between satisfiable and unsatisfiable instances, we would expect neural activity to differ.

It is likely that satisfiability has an effect on how people solve the task. For example, there may be differences in how (and when) people decide to stop deliberating based on this property of instances. As the reviewer states, this would have an effect on the time of deliberation. However, this is not something we were able to investigate with the data we collected given that our experimental design was optimized for the fMRI analysis (in particular, it was not self-paced).

2) Just about brain mapping?
The study is framed mostly as a brain mapping study: what regions correlate with typical case complexity and satisfiability? There is a bit of connectivity (PPI and Granger) but the results are not extremely informative. I would encourage the authors to motivate more their approach and the conclusions that we can draws from it. The main conclusions of the paper seem to be related to several forms of reverse inference. However, it is often the case that many concepts or task factors or effects are associated with activity in a given brain region. This is particularly a concern for variables whose neural correlate is expected to not be very specific, e.g. when looking for regions (l. 220) “linked to monitoring of uncertainty” (which is expected to also typically reflect other things such as effort, difficulty, attention). To be clear, the comment is not about new analyses, but improving a bit the framing of the paper; it could improve it significantly.

We thank the reviewer for this point. Our study is based on a novel theoretical framework, drawing on computational complexity theory, that characterises the computational hardness (computational resource requirements) of instances of the 0-1 knapsack problem purely in terms of structural properties of those instances. We have previously shown in a behavioural study that these structural properties predict both decision time and performance in the task (see in particular Franco, Bossaerts and Murawski, *J Math Psych*, 2021). (We have also shown that similar structural properties in other tasks predict human behaviour in a similar way; see Franco, Doroc, Bossaerts and Murawski, *Sci Rep*, 2022.)
The main purpose of the present study was to characterise the set of brain regions in which activity is related to those structural properties, and how activity in those regions is related to each other. To the best of our knowledge, it is the first to relate structural properties of instances of computational problems that are related to computational hardness, to neural responses, which, we believe, is an important contribution to understanding how the brain adapts to different levels of computational hardness.

Given the exploratory nature of our study, we report several of these correlates and propose mechanisms, such as uncertainty encoding, that could explain these results. However, we acknowledge that further work is required to fully characterize the dynamic neural processes involved in complex problem-solving and computational resource allocation.

We acknowledge the reviewer's concern about reverse inference, e.g., in relation to comments about uncertainty encoding. Reverse inference was never our intention. Instead, we were trying to make sense of the activation patterns we observed. To this end, we selected regions of interest (ROIs) based on previous literature linking these brain regions to cognitive processes, including the selection of the CON due to its involvement in several cognitive processes, such as uncertainty encoding and cognitive control. The ROI selection was partly based on the hypothesis that if a network for probabilistic uncertainty encoding exists in the brain, it would likely overlap with one to encode reliability (epistemic uncertainty operationalized via proof hardness/satisfiability). We tested this hypothesis by examining whether the neural correlates of satisfiability overlap with the CON, which they did. This supports our hypothesis, but we agree that there could be many other explanations. We do not claim that activity in the CON unequivocally implies uncertainty encoding. We have included this caveat in the discussion:

> *"In line with our conjecture, we found that the neural markers of satisfiability overlap with regions that encode probabilistic uncertainty. This suggests that reliability and uncertainty might constitute analogous constructs that are encoded similarly across tasks and that could serve a generic role in decision-making.* ***While our findings provide a potential explanation for the observed neural activity, further work would be needed to categorically connect these constructs across tasks. The CON is associated with a multitude of cognitive processes, and the activity we observed could be linked to any number of these. Therefore, our interpretation, while supported by our data, should not be seen as definitive. Further research is necessary to connect these constructs across various tasks definitively and to explore other potential explanations for the observed neural activity.***" [line 462]

Addressing the concern about the relevance of the connectivity analysis, we appreciate the reviewer's insights and have accordingly reduced the prominence of this aspect in our paper. We have moved most of the PPI and Granger analysis to the supplementary section, leaving a concise summary and improved motivation and main findings of these analyses in the main text. We have replaced these figures with the ROI analysis that examines the interaction between complexity metrics and accuracy, which was previously in the supplementary material. We believe this provides a more informative overview of the overall effects of computational hardness on neural activity, which follows more naturally from the key neural correlates analyses explored in the manuscript (i.e., correlates of TCC and satisfiability).

3) Put more behavioral results in the main text

I would recommend putting the appendix C.1 in the main text for two reasons. First, it introduces the measure of "instance complexity" which is advantageously defined at the instance level, whereas TCC is defined for a group of (randomly selected) instances. Second, it offers a richer

description of the data, in particular, Fig 7 shows the increase in performance with instance complexity (as the name does not suggest, instance complexity is smaller when the problem is more difficult to solve), and the absence of effect of satisfiability. I understand that this figure replicates the results of a previous paper, but it gives more confidence about the behavioral effects in this specific study.

Please see our response to comment 4 below.

4) fMRI analysis
The task design and analysis are essentially categorical. In the case of complexity, would not it be more powerful to do a regression analysis of fMRI signals with the instance complexity (defined for each instance, appendix C) rather than with high/low TCC (which, in addition to being binarized, is defined only at the level of a group of randomly sampled instance)?

In response to comments 3 and 4, we appreciate the suggestions to include more behavioral results in the main text and the potential use of instance complexity (IC) for the fMRI analysis. We understand the potential benefits of these analyses. However, our choice to base our analysis on typical case complexity (TCC) instead of IC was guided by our intention to identify neural correlates of computational hardness that do not require any knowledge of an instance's solution. TCC, unlike IC, can be computed without solving an instance, making it a more suitable choice for our study. In contrast, IC requires solving not just the instance under consideration but also solving the corresponding optimization variant (computation of optimal value obtainable with a given set of items and the weight constraint). Moreover, although IC does offer certain advantages over TCC in terms of complexity characterization, it has not been as extensively studied in the computer science literature. We have now elaborated on this rationale in the manuscript (Appendix D).

Despite the considerations above, we have analysed the BOLD data in terms of IC and have included the results in the supplementary material. Importantly, we found that the neural correlates of IC were qualitatively similar to those of TCC. For instance, no significant clusters were found during period S1 for either TCC or IC. During period S2, two clusters correlated with IC, both of which had equivalent clusters in the TCC contrast. Period S3 revealed a contrast in the posterior cingulate with IC, but this did not correspond to any cluster in the TCC results. During period S4, the neural correlates of IC partially overlapped with those of TCC, including overlap on key regions such as the right Angular gyrus, the right Anterior Insula, and the dorsal anterior cingulate cortex (dACC).

It is worth noting, however, that there were notable differences between TCC and IC contrasts, particularly in the size of the clusters, with TCC clusters tending to be larger. Additionally, several TCC clusters in periods S2 and S3 were not identified when using IC, a discrepancy likely due to the statistical power. Our study was designed to optimize power for detecting the categorical effects of TCC and satisfiability, rather than the parametric modulation effect of IC.

Regarding the suggestion to move Appendix C.1 to the main text, we would prefer not to do this as it would require a considerable amount of additional explanation of concepts and models. Given that IC is only marginally relevant to our manuscript, we believe its inclusion in the main text could potentially introduce unnecessary complexity and confusion, particularly considering the additional mathematical definitions that are not then used for the core neuroimaging analysis. We hope this clarifies our methodological choices and the rationale behind them.

Minor:
- l. 138: A formalism is introduced to define constrainedness and satisfiability, but the formalism is not very useful because it is not explained. What is p, c, v_i, w_i, N? I think I guessed but it would be better to have them explained. I would recommend to either remove the corresponding section of the results (that explains the method) and simply claim that you have a formalism for constrainedness and satisfiability, or to explain it in a way that is self contained (instead of referring the reader to you previous paper, Franco 2021). If such a section is long, then it could appear in the Method section. Currently, the Method section does not explain how constrainedness and satisfiability are computed. Section 4.4 of the Method is also impossible to understand without having been introduced with the formalism of alpha_p and alpha_c.

In response to the reviewer's minor concern regarding the formalism introduced on line 138, we have made comprehensive revisions to both the results and methods sections. We have defined all terms explicitly and expanded the methods section to provide a detailed explanation of how we compute constrainedness and satisfiability, thereby reducing the need for readers to refer to our previous work. Furthermore, we have revised Section 4.4 of the Methods and Materials section to introduce the formalism of alpha_p and alpha_c in a more accessible manner.

- The paper repeatedly reports an absence of progression during the task (e.g. no change in performance as the number of trials increases). However, the confidence interval is close to detecting an effect, with 0 (no effect) being very close to the lower bound of the interval. Could it be that the change over trials is not linear but gradually saturates, and would be better captured by a regression with the square-root of the number of trials?

In response to the concern raised regarding the potential non-linear progression during the task, we appreciate the reviewer's suggestion to consider a regression model with the square root of the number of trials. To address this, we implemented three models: a linear model, a model with a square-root transformation of trials, and a model with a quadratic transformation.

To determine the best fit, we compared these models using the Expected Log Pointwise Predictive Density (ELPD), a measure of model performance. Our analysis revealed that the linear model, albeit marginally, provided the best fit for our data. Therefore, we have decided to retain this model in the manuscript.

Model (transformation of trial number variable)	ELPD

Linear Model	-546.65
Suared Root Model	-546.96
X Squared Model	-547.27

However, we acknowledge the reviewer's point that a saturation effect cannot be entirely ruled out. In fact, the model with a square-root transformation of trials did show a positive, albeit non-significant, effect of trial progression on accuracy (beta= 0.08, CI_95%=[-0.02,0.17]). This result suggests that there may be some degree of saturation, but the effect is not strong enough to significantly improve the model fit over the linear model. We hope that this explanation provides a clear and satisfactory response to the reviewer's query.

- l. 189: "We found that the neural correlates of TCC varied throughout the duration of the solving stage (Fig 2a, Table 1)." It is difficult to see some dynamics in this figure because maps are thresholded, therefore some small changes in the effect can have dramatic effects in the figure (e.g. when crossing the threshold for significance). I would recommend using unthresholded maps for this figure (together with Table 1 which reports significance levels corrected for multiple comparisons).

In response to the reviewer's comment regarding line 189 and the difficulty in discerning dynamics due to thresholded maps, we appreciate the suggestion to use un-thresholded maps. However, we would like to point out that un-thresholded maps often present challenges in visualization due to the variability in effect estimates across the entire brain. To navigate this issue effectively, an interactive plot would be more appropriate. In line with this, we have supplemented our material with two HTML files that contain unthresholded contrasts for both Fig TCC and Fig Satisf. These additional files will allow interested readers to interact with the plot and choose their preferred viewpoint of the brain. This interactive approach should provide a more comprehensive understanding of the data, moreover, as part of the data being shared upon publication, we include full brain maps of each of the contrasts analyzed.

<u>Reviewer #2:</u>

This study uses a formal definition of computational complexity to examine the neural mechanisms of complexity processing in humans. While being scanned in a 7T MRI, participants engaged in a decision making task that dissociates problem complexity from choice difficulty. The results implicate a network of interacting brain areas including dorsal anterior cingulate cortex, the anterior insula and the angular gyrus.

The study's major strength is its formal approach to studying complexity. The fMRI analyses are appropriate and relatively straightforward. Overall I believe that this paper will make an important contribution to the literature. That said, in my view the main ideas are described rather

abstrusely, especially for a non-expert like me. The paper quickly serves up a word salad of terms such as NP-complete, NP-hard, NP, complexity, proof hardness, random ensembles of instances, typical-case complexity, satisfiability, co-NP-complete, reliability, and constrainedness. I imagine that these concepts may be familiar to many computer scientists, but for an interdisciplinary journal more could be done to introduce this terminology. My comments below reflect this opinion.

1. Complexity is measured using “random ensembles of instances”. This technique seems to have been applied mainly by the authors themselves in a few of their recent studies, in order to derive what they call “typical-case complexity”. Given that this technique is not yet widely used in neuroscience, it should be described here in greater depth.

We thank the reviewer for the valuable feedback and recognition of our study's strengths, particularly in our formal approach to studying complexity and the appropriateness of our fMRI analyses. We appreciate the reviewer’s perspective on the complexity of the terminology used in our paper, and we acknowledge that the jargon may be challenging for those not familiar with theoretical computer science.

In response to the reviewer’s comment about the use of "random ensembles of instances" to measure complexity, we agree that this technique, which we have employed in our recent studies to derive "typical-case complexity" (TCC), may not be widely recognized in the neuroscience field yet. We understand the need for a more comprehensive explanation, given its novelty and the interdisciplinary nature of the journal.

Therefore, we have taken the reviewer’s suggestions into consideration and have expanded upon the description of TCC. We have endeavored to present the concept with greater clarity in the results section (section 2.1.2) and further elaborated on it in the methods section (section 4.4). Our aim is to make this technique more accessible and understandable to a broader audience, including those outside the realm of computer science.

2. Brief definitions of NP-complete and co-NP-complete are warranted, as well as descriptions of how these terms relate to NP-hard and NP problems.

We have taken the reviewer’s valuable feedback into account and have made revisions to our manuscript to ensure a clearer presentation for an interdisciplinary audience. Specifically, we have reduced the use of highly specific terminology in the introduction (removed co-NP-complete, NP-hard and NP) and have expanded the methods section to provide more detailed explanations of the terms 'NP-complete' and 'co-NP-complete', as well as their relationship with 'NP-hard' and 'NP' problems. We've also elaborated on how these terms interrelate with proof hardness and typical case complexity (TCC). To further facilitate comprehension, we have incorporated a new figure in the methods section (Figure 5), and we have updated the main behavioral results figure in the main text. These figures visually represent these complex concepts, aiding in the reader's understanding. We believe these changes will make the paper

more accessible and comprehensible to a wider audience, including those who may not have a background in computer science.

3. Constrainedness is defined according to equations for alpha-p and alpha-c (line 138), but the parameters in these equations are undefined: what are p, v, I, c and w?

We now clearly define all the parameters, namely p, v, I, c, and w, in the main body of the text.

4. Figure 1b indicates that this task is characterized by essentially four types of computational problems: 1) low TCC and satisfiable (“underconstrained”, low complexity); 2) low TCC and unsatisfiable (“overconstrained”, high complexity), 3) High TCC and satisfiable (medium complexity); and 4) high TCC and unsatisfiable (medium complexity). It would be helpful if an example trial were provided for each of these four conditions. I am particularly interested in the high complexity trials, which are both low typical-case complexity and unsatisfiable. What is it about these trials that make them overconstrained and therefore easy to solve, despite being unsatisfiable? Further, why is it not possible to have low complexity trials that are unsatisfiable and high complexity trials that are satisfiable? An example for each of the 4 conditions would go a long way toward making the entire paper more transparent.

We appreciate the reviewer's observation regarding the need for clarity in the explanation of the four types of computational problems presented in our study. To clarify, our study explores two theoretically orthogonal dimensions of computational difficulty: proof hardness (operationalized as satisfiability) and complexity (operationalized as typical case complexity, TCC). Our experiment was designed as a 2x2 balanced design, ensuring that each participant was presented with an equal number of instances (trials) for each condition. This resulted in four types of instances:

1. High TCC and satisfiable (underconstrained),
2. High TCC and unsatisfiable (overconstrained),
3. Low TCC and satisfiable (underconstrained), and
4. Low TCC and unsatisfiable (overconstrained).

Indeed, there are high complexity instances that are satisfiable (category 1) as well as low complexity instances that are unsatisfiable (category 4). The latter instances, being overconstrained, are characterized by low computational difficulty. This may seem counterintuitive, but a plausible explanation lies in the fact that when the search space is highly constrained, large portions of it can be excluded from the search through brief logical rules. For example, if the target profit exceeds the sum of all item values, the solver can quickly determine the instance is unsatisfiable, as even including all items in the knapsack wouldn't meet the profit constraint.

To enhance the clarity of our study, we have made two significant amendments. Firstly, we have revised Figure 1B, which previously suggested three levels of complexity onto which TCC and satisfiability mapped. We clarified that there are only two levels of complexity (TCC) and two of

proof complexity (satisfiability). Secondly, in line with the reviewer's suggestion, we have included an example to explain TCC (lines 139-148). These amendments should provide a clearer understanding of our study's design and findings.

5. Regarding the fMRI results, can the authors compare the underconstrained vs. overconstrained conditions? Are these results different from the satisfiable vs. unsatisfiable contrast, given that the high TCC trials are removed from the comparison?

We present the results of the contrast suggested below. We find that this contrast shows a few differences from the satisfiability analysis presented in the manuscript. On the one hand, and most importantly, we see a significantly lower number of significant clusters. This is likely due to a reduction in power, since the number of trials is reduced to a half of the original dataset when restricting instances to low TCC. On the other hand, three additional clusters are found:

1. A cluster in the left frontal pole on period S3.
2. A cluster in the left occipital superior cortex on period S4.
3. A cluster in the left Angular Gyrus (AG) pole on period S4.

The cluster in the AG is explained in Figure 3.C in the main text. This figure shows that BOLD in the AG shows a marked decrease in activation specifically in satisfiable instances with low TCC compared to the other conditions. We speculate that a similar activation pattern could explain the other two clusters found.

Overall, we advise caution when interpreting these results, given the marked reduction in power. As such, we did not include these results in the manuscript.

Table 8: **Satisfiability clusters for low TCC instances**. Significant cluster-wise FWE-corrected ($p < 0.05$) clusters (using an uncorrected threshold of $p < 0.001$) from the *Unsatisfiable-Satisfiable* contrast on only instances with low TCC. Coordinates are in MNI space.

			Cluster statistics			Peak statistics			
Stage	Region	Side	Volume(mm^3)	β_{mean}	SEM	β_{peak}	x	y	z
S1	MFG	RH	1110.0	-0.24	0.003	-0.38	45	33	18
	Frontal Pole	RH	671.7	-0.20	0.003	-0.33	30	62	-8
	Supramarginal gyrus	RH	606.2	-0.19	0.002	-0.26	42	-47	45
	Caudate	LH	553.0	-0.25	0.004	-0.35	-8	2	-1
S3	Frontal Pole	RH	1577.0	-0.19	0.002	-0.28	13	57	31
	SFG	LH	1191.9	-0.23	0.003	-0.35	-18	34	56
	Frontal Pole	LH	1097.7	-0.20	0.003	-0.31	-21	39	36
	Anterior cingulate	LH	864.3	-0.29	0.005	-0.47	-3	52	4
S4	AI	RH	1327.1	0.31	0.003	0.48	32	28	0
	Occipital superior cortex	LH	847.9	0.29	0.005	0.44	-24	-65	40
	AG	RH	815.1	-0.26	0.003	-0.37	54	-63	42
	AG	LH	778.2	-0.33	0.003	-0.44	-46	-70	44

Clusters Period S1:

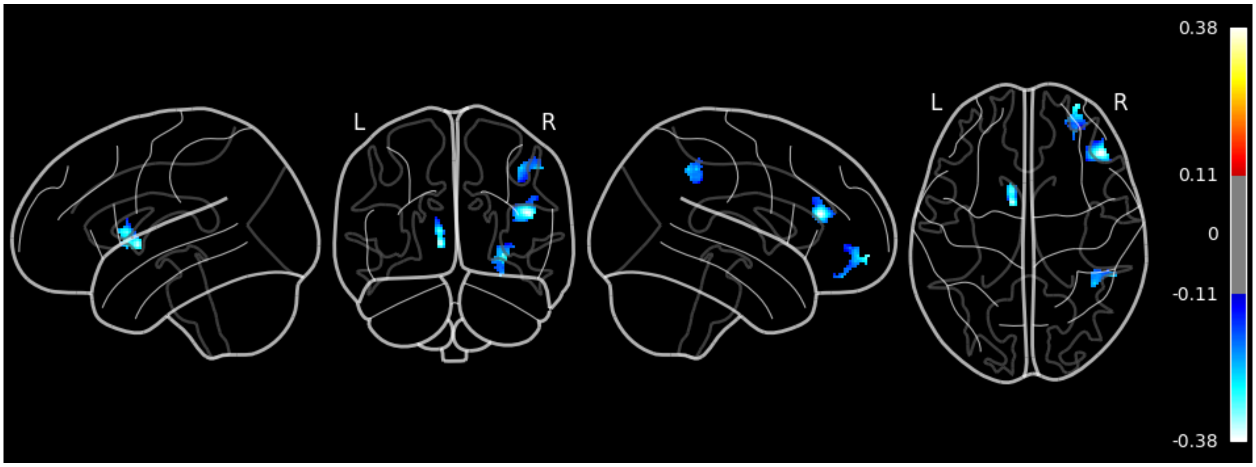

Clusters Period S3

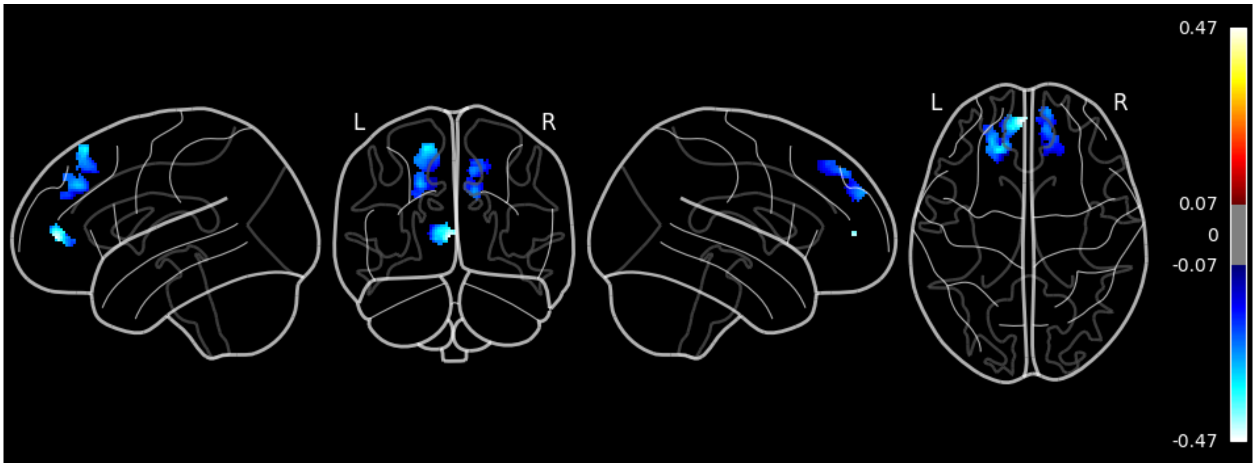

Clusters Period S4

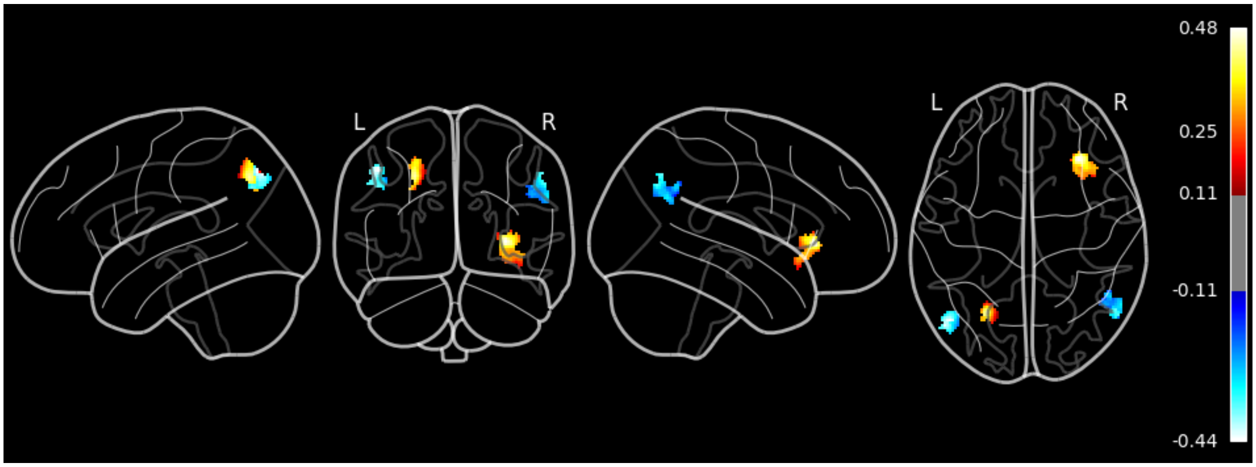

6. Can the authors speculate more about the cognitive processes involved in the task? It is a bit difficult to understand what is going on without some kind of theoretical model to anchor the data on. To be sure, these issues are touched on in the paper — for example, it states that "TCC can be potentially estimated from early on in the solving stage without the need to know the solution to the problem" (line 406), and "evidence toward a solution can be accumulated faster in low TCC compared to high TCC instances (line 425) (see also lines 437-448) — but in my view these ideas could be fleshed out even more. How can people estimate TCC? And depending on TCC level, what strategies do they apply, and does it depend on the level of complexity?

For example, presumably at the start of each trial subjects use some kind of general purpose strategy, whereas later in the trial they tailor or switch strategies according to the specific problem type. Most interesting is that although performance is relatively good on all low TCC trials, the hemodynamic response suddenly decreases for the satisfiable (underconstrained) trials but not the unsatisfiable (overconstrained) trials. Am I right to assume that although accuracy is about the same for both underconstrained and overconstrained trials, RTs are much slower for the overconstrained relative to the underconstrained trials? These are issues that a processing model could help illuminate. (I suggest that Figure 1B also show the RTs in addition to accuracy).

We appreciate the reviewer's interest in the cognitive processes involved in the task and the suggestion for a more detailed theoretical model. The crux of our paper is the exploration of problem-solving in a complex task without the necessity to identify specific cognitive strategies employed by individuals. This approach is particularly beneficial in complex tasks where an increase in complexity often corresponds to an increase in the diversity of available problem-solving strategies. Previous research, including our own (Murawski, C., Bossaerts, P., 2016), has demonstrated that the strategies individuals utilize to solve the knapsack problem can vary significantly not only between individuals but also between instances and trials.

In this mansucript, we introduce a methodology that focuses on the intrinsic characteristics of a complex computational task, allowing us to identify neural activation patterns that are related to intrinsic computational hardness. This approach is akin to those used in the study of other task types. For example, in perceptual tasks, the neural underpinnings have been explored by studying the differences in neural processes relative to intrinsic task features. In probabilistic tasks, invariants are captured by examining the neural processes linked to intrinsic characteristics such as mean, variance, and related stochastic metrics.

Applying this framework to the study of complex tasks, we identified neural invariants of problem-solving in a generic manner. Like in perceptual and probabilistic tasks, the relevant features (e.g., computational difficulty) can be studied across tasks without complete knowledge of the strategies employed. We hope that this work will set the foundations to build elaborate process models. As such, we have made four amendments to the text to provide potential explanations for the cognitive processes involved. We hope that these changes will offer a more

comprehensive understanding of the cognitive processes at play in complex problem-solving tasks.

- “For example, if an individual predicts that a given instance could potentially be satisfiable, they could search for a subset of items that meets the defined constraints. However, if the perception is that the given instance might be unsatisfiable, then the individual may start a search for proof of infeasibility, like verifying (in an extreme case) whether fitting even a single item into the knapsack is impossible due to all the item weights surpassing the allowed weight capacity. However, this account would still require participants to implement different strategies” (line 483).
- “Indeed, TCC could be estimated by performing a straightforward summation followed by a division (add all item values and divide the result by the target profit). As such, we expected to see neural correlates of TCC from early on in the trial.” (line 433).
- Moved to main text from appendix: “One possible explanation for this is attentional engagement on the task. If a participant does not actively engage in the task, they are more likely to have an incorrect solution. In turn, the likelihood of reaching an incorrect answer due to inattention is higher among instances with low computational difficulty.” (line 503).
- “Arguably, evidence toward a solution can be accumulated faster in low TCC compared to high TCC instances. This follows directly from the theoretical prediction that the time needed to reach the solution is, on average, higher for instances with high TCC than low TCC. The difference in rate accumulation would imply that regions that encode evidence accumulation would show a higher activation on low TCC instances early in the trial…” (line 452).

Regarding the reaction times (RTs) for the decision knapsack problem, unfortunately, such data is not available in our study since the task was not self-paced.

7. In my view the results of the PPI/Granger causality analyses are not very informative. So far as I can see, the analyses show that three somewhat arbitrary ROIs interact with each other as a function of task complexity. Mightn’t we have guessed this going into the experiment? What have we learned? Whether or not these analyses should remain in the paper is a judgement call, but I’d advocate for removing them.

In response to the reviewer’s comment regarding the perceived limited informativeness of the PPI/Granger causality analyses, we appreciate your perspective and have taken it into consideration. While these analyses might seem to merely confirm the interaction of three Regions of Interest (ROIs) as a function of task complexity, we believe they do provide valuable insights into the intricate neural dynamics underpinning the problem-solving process. However, we understand that the perceived value of these analyses may vary among readers. As such, following the reviewer’s suggestion, we have decided to relocate the bulk of the connectivity analysis to the supplementary material. This move allows interested readers to delve into the details if they wish while maintaining the main body of the paper more streamlined and focused. In the revised manuscript, we now only provide a succinct overview of the motivation, results, and implications of the connectivity analyses. We believe this adjustment strikes a balance

between preserving the comprehensiveness of our study and ensuring the readability and coherence of the main text.
