# Supplementary material for: The neural dynamics associated with computational complexity
Source: PLoS Comput Biol. 2024 Sep 23;20(9):e1012447. doi: 10.1371/journal.pcbi.1012447 (PMC11449275; doi:10.1371/journal.pcbi.1012447)
Supplement: Attachment — Submitted filename: response_letter.pdf [file pcbi.1012447.s003.pdf]

Dear Editor,

Thank you for considering our revised manuscript, “The neural dynamics associated with computational complexity" (PCOMPBIOL-D-23-01530) for publication in PLOS Computational Biology. We would like to also thank the reviewers for their thoughtful and constructive comments, which have helped us improve the manuscript. We have addressed all of the issues raised by the reviewers in the attached revisions. All reviewer comments are below in black with our comments in blue.

Reviewer #1:

I thank the authors for their response which address my comments.

I would recommend that they screen their paper for acronyms that are not defined on their first occurrence (if defined at all). For instance, a number of brain regions are not defined beyond their acronyms: CON, dACC, IPS, etc.

We thank the reviewer for highlighting this issue. In response, we have now defined all previously undefined acronyms and redefined all acronyms in the Methods section. To ensure clarity in the Methods section, we have provided these definitions both in the main text and in Table 1.

Reviewer #2:

The authors have done a good job of addressing my concerns about the previous version of the manuscript. However, I still have a few remaining concerns. The importance of these issues hovers somewhere between major and minor.

1. The authors state that they cannot analyze RT because the task is not self-paced. I do not follow this logic: RTs are typically analyzed in speeded reaction time tasks that have a maximum response time threshold. So why not look at them? The reason that this is important is because RTs provide an independent measure of trial difficulty.

We appreciate this suggestion. Our experimental protocol was specifically designed with the neuroimaging analysis of the solving stage as the primary focus. We kept the duration of this stage constant for each participant and trial, to keep the number of samples for this stage in the trial constant. We concur that complexity metrics such as TCC and satisfiability likely influence deliberation time (cf. e.g., Franco et al., 2022 [10]). Validating this relation was not the primary aim of our current study, however.

We don’t have any reason to believe that RTs during the (separate) response stage are a measure of difficulty.

We do acknowledge the importance of the impact of TCC and satisfiability on the decision-making process and deliberation time, and have now highlighted it in the discussion section for potential exploration in future research:

*“Contrary to our expectations, we only found significant clusters in the CON starting from the third period of the solving stage. These might reflect markers of expected performance, but other explanations cannot be excluded. For instance, this effect might reflect differences in time-on-task between TCC conditions [45]****; indeed, previous work has shown that TCC affects time-on-task in other computational problems [10]****. This explanation, however, would still allow these activation patterns to represent differences in neural markers such as reliability and expected performance. This follows from the fact that time-on-task is an endogenous variable of the system. That is, the agent decides when to stop reasoning about the problem, and as such, this decision would likely follow from a subjective belief on how well they can expect to perform given the current candidate solution. Therefore, differences in time-on-task between high and low TCC instances may be related to differences in subjective beliefs of both expected performance and reliability.* ***Future work is needed to explore how and when people decide to stop deliberating, as well as the role of computational difficulty in this process****.”*

2. Although the terminology and task parameters are much clearer now, I still don’t fully grok the basic idea. On the one hand, proof hardness characterizes the complexity of verifying that a solution to a problem is correct. Unsatisfiable problems have high proof hardness, which because they require verifying that no witnesses exist, might require more than polynomial time. Therefore these trials should be difficult. On the other hand, unsatisfiable problems with low typical case complexity can be solved by applying logical rules. In other words, these trials are easy. So which is it–easy or difficult? I guess the operating word here is “might” (require more than polynomial time). I really believe that the paper will more impactful if the authors address this issue concretely.

We appreciate the reviewer's observation regarding the need for clarity in explaining the four types of instances presented in our study. To provide further clarity, our study investigates two theoretically orthogonal dimensions of computational difficulty: proof hardness (operationalized as satisfiability) and complexity (operationalized as typical case complexity, TCC).

Unsatisfiable instances with low TCC are "easy" to solve but candidate solutions are "hard" to verify. While these two metrics, TCC and proof hardness, may be related, they exist on separate dimensions and can be studied independently, which is why the experiment was designed using a 2x2 factorial approach. We currently do not have a theoretical prediction on how these dimensions might interact, so we cannot consolidate them into a single dimension of computational difficulty.

To address the reviewer's concern, we have added a figure in the Materials and Methods section (Fig. 6) to illustrate the 2x2 design and clarify the computational difficulty of instances in each category. This figure explicitly shows how instances hold distinct profiles of computational difficulty, including cases that are easy to solve but hard to verify. We hope this addition enhances the clarity of our explanation.

3. Relatedly, the authors write in the cover letter (point 4) that they provided an example of the task on lines 139-148, but I can’t find any example at these lines.

Apologies, the line numbers were incorrect. Here is the text added in relation to the example used to clarify the definition of the TCC metric:

“As an illustration, consider the instance presented in Fig 1a. If the target profit were set to a large value (e.g., $300), the instance would be overconstrained (it is likely that no combination of items satisfies both constraints), whereas if it were set to a very low value (e.g., $20), the instance would be underconstrained. The more extreme these values are, the easier the instance is to solve”
